# Clinical Strategies Against Early Hematoma Expansion Following Intracerebral Hemorrhage

**DOI:** 10.3389/fnins.2021.677744

**Published:** 2021-08-30

**Authors:** Kanta Tanaka, Kazunori Toyoda

**Affiliations:** Department of Cerebrovascular Medicine, National Cerebral and Cardiovascular Center, Suita, Japan

**Keywords:** intracerebral hemorrhage, hematoma enlargement, blood pressure lowering, anticoagulant reversal, hemostatic therapy, recombinant activated factor VII

## Abstract

Hematoma volume is the strongest predictor of morbidity and mortality after intracerebral hemorrhage. Protection against early hematoma growth is therefore the mainstay of therapeutic intervention for acute intracerebral hemorrhage, but the current armamentarium is restricted to early blood pressure lowering and emergent reversal for anticoagulant agents. Although intensive lowering of systolic blood pressure to <140 mmHg appears likely to prevent hematoma growth, two recent randomized trials, INTERACT-2 and ATACH-2, demonstrated non-significant trends of reduced hematoma enlargement by intensive blood pressure control, with only a small magnitude of benefit or no benefit for clinical outcomes. While oral anticoagulants can be immediately reversed by prothrombin complex concentrate, or the newly developed idarucizumab for direct thrombin inhibitor or andexanet for factor Xa inhibitors, the situation regarding reversal of antiplatelet agents is not yet quite as advanced. However, considering at most the approximately 10% rate of anticoagulant use among patients with intracerebral hemorrhage, what is most essential for patients with intracerebral hemorrhage in general is early hemostatic therapy. Tranexamic acid may safely reduce hematoma expansion, but its hemostatic effect was insufficient to be translated into improved functional outcomes in the TICH-2 randomized trial with 2,325 participants. In this context, recombinant activated factor VII (rFVIIa) is a candidate to be added to the armory against hematoma enlargement. The FAST, a phase 3 trial that compared doses of 80 and 20 μg/kg rFVIIa with placebo in 841 patients within 4 h after the stroke onset, showed a significant reduction in hematoma growth with rFVIIa treatment, but demonstrated no significant difference in the proportion of patients with severe disability or death. However, a *post hoc* analysis of the FAST trial suggested a benefit of rFVIIa in a target subgroup of younger patients without extensive bleeding at baseline when treated earlier after stroke onset. The FASTEST trial is now being prepared to determine this potential benefit of rFVIIa, reflecting the pressing need to develop therapeutic strategies against hematoma enlargement, a powerful but modifiable prognostic factor in patients with intracerebral hemorrhage.

## Introduction

Therapeutic strategies for intracerebral hemorrhage lag a long way behind those for acute ischemic stroke (Toyoda et al., 2016). A systematic review including 36 eligible studies with a mid-year range from 1983 to 2006 showed a stable case fatality rate of around 40% during these decades, indicating delays in the development of effective treatments for acute intracerebral hemorrhage (van Asch et al., 2010). If the hematoma remains small, brain damage should be minor and the patient prognosis would also be good. A strategy we can implement for intracerebral hemorrhage in general to achieve the goal of minimizing hematoma size is early blood pressure lowering, but the evidence for its effects on hematoma volume control and clinical outcome remain ambiguous (Anderson et al., 2013; Qureshi et al., 2016). The treatment for acute intracerebral hemorrhage analogous to reperfusion therapy for acute ischemic stroke would be early hemostatic therapy. However, its development has yet failed to see the light of day (Mayer et al., 2008; Sprigg et al., 2018; Meretoja et al., 2020). From a clinical perspective, this review outlines the current therapeutic strategies and future prospects for protection against early hematoma expansion for reducing brain damage and improving patient outcomes after acute intracerebral hemorrhage.

## Hematoma Volume: A Key Player in Brain Damage Ever Since Establishment of the Entity

The most important fact that should be emphasized when considering treatments to improve the prognosis of patients with intracerebral hemorrhage is that hematoma volume is the strongest predictor of morbidity and mortality after intracerebral hemorrhage. According to one observational study reported in 1993, patients with a hematoma volume >60 mL showed 30-day mortality rates of 93% for deep hemorrhages and 71% for lobar hemorrhages. For hematoma volumes of 30–60 mL, mortality rates were 64% for deep hemorrhages and 60% for lobar hemorrhages. In patients with hematoma volume <30 mL, 30-day mortality rates were 23% for deep hemorrhages and 7% for lobar hemorrhages. In addition, only 1 of the 71 patients with a hematoma volume >30 mL could function independently at 30 days (Broderick et al., 1993). Such a clear impact of hematoma volume on mortality and morbidity has been confirmed in multiple cohorts and, given that the initial pathological damage to the brain from intracerebral hemorrhage is due to mechanical compression caused by the hematoma, the strong association between hematoma volume and patient prognosis is rational (Portenoy et al., 1987; Daverat et al., 1991; Tuhrim et al., 1991; Juvela, 1995; Qureshi et al., 1995; Hallevy et al., 2002). In addition to the degree of mechanical compression into the surrounding brain tissue, secondary brain damage due to inflammation, oxidative stress, or cytotoxicity due to erythrocyte lysates also becomes severe when hematoma volume is large (Duan et al., 2016; Wan et al., 2016; Zhang et al., 2017; Shao et al., 2019). At present, however, clinically applicable therapeutic agents for such secondary brain damage are unavailable (Hemphill et al., 2015). Keeping the hematoma small is therefore a key strategy to protect the surrounding normal brain (Table 1).

**TABLE 1:** Current status of potential strategy for preventing hematoma expansion in patients with acute intracerebral hemorrhage (ICH).

**Blood pressure lowering**
**INTERACT2 trial (Absolute difference in hematoma growth = −1.4 mL by intensive treatment;**Anderson et al., 2013) -Intensive treatment with target SBP level of <140 mmHg or standard treatment with a target SBP of <180 mmHg -Improvement in functional outcomes with intensive lowering of blood pressure
**ATACH-2 trial [Rate difference of hematoma expansion (>33% increase) = −5.5% by intensive treatment]** (Qureshi et al., 2016) -Intensive treatment with a target SBP level of 110–139 mmHg or standard treatment with a target of 140–179 mmHg -No improvement in functional outcomes with intensive blood pressure lowering
**Emergent reversal of anticoagulant agents**
**VKA** -Patients with ICH whose INR is elevated because of VKA should have their VKA withheld, receive therapy to replace vitamin K-dependent factors and correct the INR, and receive intravenous vitamin K (**Class I; Level of Evidence C; AHA/ASA Guideline 2015**; Hemphill et al., 2015) -PCCs may have fewer complications and correct the INR more rapidly than FFP and might be considered over FFP (**Class IIb; Level of Evidence B; AHA/ASA Guideline 2015**; Hemphill et al., 2015)
**Heparin** -Protamine sulfate may be considered to reverse heparin in patients with acute ICH (**Class IIb; Level of Evidence C; AHA/ASA Guideline 2015**; Hemphill et al., 2015)
**Dabigatran: RE-VERSE AD (Idarucizumab;**Pollack et al., 2017) -Idarucizumab for patients who had uncontrolled bleeding or were about to undergo an urgent procedure -Median time since last intake of dabigatran, 15.6 h -The median maximum percentage reversal of dabigatran was 100% on the basis of either the diluted thrombin time
**Factor Xa inhibitors (apixaban, rivaroxaban, and edoxaban): ANNEXA-4 (Andexanet**; Connolly et al., 2019) -Andexanet for patients with acute major bleeding within 18 h after administration of a factor Xa inhibitor −82% of patients had excellent or good hemostatic efficacy at 12 h, with the rate for thrombotic events of 10%
**Platelet transfusion for ICH associated with antiplatelet use**
**PATCH trial (Absolute difference in hematoma growth = +0.85 mL by platelet transfusion;**Baharoglu et al., 2016) -Platelet transfusion vs. standard care for acute ICH associated with antiplatelet therapy use -Death or dependence was more frequent in the platelet transfusion group
**Early hemostatic therapy**
**Tranexamic acid** **TICH-2 trial (Absolute difference in hematoma growth = −1.37 mL by tranexamic acid treatment**; Sprigg et al., 2018) **STOP-AUST trial (Absolute difference in hematoma growth = −1.8 mL by tranexamic acid treatment**; Meretoja et al., 2020) -The hemostatic effects would be insufficient to be translated into improved functional outcomes
**Recombinant activated factor VII (rFVIIa)** -Although rFVIIa can limit the extent of hematoma expansion in non-coagulopathic ICH patients, there is an increase in thromboembolic risk with rFVIIa and no clear clinical benefit in unselected patients. Thus, rFVIIa is not recommended (**Class III; Level of Evidence A; AHA/ASA Guideline 2015**; Hemphill et al., 2015)
**Absolute difference in hematoma growth in FAST trial** (Mayer et al., 2008) **20** μ**g/kg rFVIIa: −2.6 mL** **80** μ**g/kg rFVIIa: −3.8 mL**


## Early Hematoma Enlargement: The Most Important Therapeutic Target

Clinical observations when computed tomographic scan was clinically applied shows that more than half of patients with acute intracerebral hemorrhage experience a gradual, smooth worsening of the neurological deficits within several hours from onset (Mohr et al., 1978; Caplan et al., 1983; Bogousslavsky et al., 1988). Such clinical data indicate the presence of a therapeutic time window from the rupture of cerebral blood vessels to hemostasis and hematoma stabilization, during which time the hematoma grows. Many observational studies using brain imaging have shown that most hematoma enlargements occur in the first 24 h, and all studies examining early hematoma growth have found the higher rates of growth within 6 h of onset (Chen et al., 1989; Kazui et al., 1996; Brott et al., 1997; Fujii et al., 1998; Jauch et al., 2006; Cucchiara et al., 2008; Toyoda et al., 2009). The rate of early hematoma growth is high and have been reported as around 20–30% within the first 24 h from onset, although the rates vary according to the definition of hematoma growth.

Patients with hematoma expansion were found to display an increased frequency of neurological deterioration (66 vs. 14%) and higher rate of intracerebral hemorrhage-related mortality (29 vs. 3%) compared to patients without expansion (Kazui et al., 1996). For every 10% increase in the hematoma volume at 24 h, the hazard ratio for mortality increased by 5% (Davis et al., 2006). Therapy directed at stopping bleeding as early as possible could thus potentially decrease mortality and improve neurological outcomes.

## Current Armamentarium Against Early Hematoma Enlargement

### Early Blood Pressure Lowering

The current armamentarium available for prevention of hematoma expansion is restricted mainly to early blood pressure lowering. Elevated blood pressure is quite frequently observed in acute intracerebral hemorrhage, with 75.0% of patients showing systolic blood pressure (SBP) ≥ 140 mmHg and 33.1% having SBP ≥ 160 mmHg (Qureshi et al., 2007). Although the elevated blood pressure spontaneously declines over the next few days, higher SBP (particularly initial SBP ≥ 200 mmHg) is associated with both hematoma enlargement and increased mortality (Kazui et al., 1997). The Second Intensive Blood Pressure Reduction in Acute Cerebral Hemorrhage Trial (INTERACT2) demonstrated marginally better functional outcomes for acute intracerebral hemorrhage patients with early intensive blood pressure lowering to a targeted SBP of <140 mmHg compared to the standard lowering to a SBP of <180 mmHg (Anderson et al., 2013). However, another randomized controlled trial, the Antihypertensive Treatment of Acute Cerebral Hemorrhage (ATACH)-2 trial, did not show benefit in terms of reductions in death or disability between the two treatment groups under the same target SBP as INTERACT2 (Qureshi et al., 2016). As high SBP theoretically leads to increased local blood pressure in the culprit ruptured artery and drives continued bleeding, the conflicting results regarding intensive blood pressure lowering and prognosis among patients with intracerebral hemorrhage has prompted research into the underlying reasons for such conflicts.

One possible mechanism is the existence of racial differences in responsiveness to blood pressure lowering therapy. In the INTERACT2, 68% of the participants were from Asia, while in ATACH-2, the percentage was 56% (Anderson et al., 2013; Qureshi et al., 2016). A regional/racial sub-analysis of ATACH-2 trial data showed that hematoma expansion was attenuated by intensive blood pressure lowering only in Asians (Toyoda et al., 2021). Characteristically, hematomas in the basal ganglia were more common in Asian patients than in non-Asian patients. The rate of basal ganglia involvement was 50.6% (506/1,000) in the ATACH-2 trial, which was higher than the 42.9% (1,215/2,829) rate in the INTERACT2 trial (Delcourt et al., 2017). As an explanation for such observations, possible differences in the relationship between systemic blood pressure and the local blood pressure among the different bleeding sites may be considered. In general, blood pressure drops as flow is divided among branches, so blood pressure is much lower at the distal end of long arteries with many branches than in the parent artery. Using computational modeling, Blanco et al. estimated pressure gradients from proximal to distal regions of the cerebral vasculature. In their model, when blood pressure in the brachial artery was 192/113 mmHg, the pressure in the arterioles of the lenticulostriate arteriolar bed would be 169/101 mmHg, whereas the pressure in the same-sized arterioles of the posterior parietal arteriolar bed was estimated as 125/74 mmHg (Blanco et al., 2017). This estimation indicates that blood pressure within the culprit vessel during the acute phase after intracerebral hemorrhage differs between the deep and lobar hemorrhages. In a sub-analysis of ATACH-2 data, intensive blood pressure lowering was associated with a decreased risk of expansion for basal ganglionic hematoma, but not for thalamic or lobar hematomas (Leasure et al., 2019).

Furthermore, an excessive reduction in SBP can increase cardiorenal adverse events, offsetting the clinical benefits of blood pressure lowering therapy on risk reduction for hematoma expansion (Toyoda et al., 2019; Fukuda-Doi et al., 2021). The achieved mean minimum SBP in the intensive treatment group in ATACH-2 was <130 mmHg, while the mean SBP in the intensive group of INTERACT2 was approximately 140 mmHg (Anderson et al., 2013; Qureshi et al., 2016). A sub-analysis of INTERACT2 showed that mean SBP of 130–139 mmHg was associated with the best outcome, whereas an increase in poor outcome was suggested for mean SBP of <130 mmHg (Arima et al., 2015). Setting a more tailored target blood pressure level according to hematoma location, bleeding etiology, or known risks for hematoma enlargement is thus increasingly being recognized as important.

### Emergent Reversal of Anticoagulant Agents

Another important treatment for preventing hematoma enlargement is a prompt reversal of the effect of anticoagulant agents. Both antiplatelets and anticoagulants increase the risk of hematoma growth, but the risk is markedly higher for those taking anticoagulants (Flibotte et al., 2004; Huttner et al., 2006; Cucchiara et al., 2008; Toyoda et al., 2009). The use of prothrombin complex concentrates should be considered as a treatment strategy for reversing anticoagulant effects in intracerebral hemorrhage patients taking warfarin (Yasaka et al., 2003; Huttner et al., 2006; Lee et al., 2006; Goldstein et al., 2015; Chai-Adisaksopha et al., 2016; Steiner et al., 2016). In recent years, the usage rates of direct oral anticoagulants have increased among patients who require oral anticoagulation. Phase III trials of direct oral anticoagulants have demonstrated lower incidences of intracranial hemorrhage or hemorrhagic stroke among patients treated with direct oral anticoagulants compared to those treated with warfarin (Connolly et al., 2009; Granger et al., 2011; Hori et al., 2012; Giugliano et al., 2013). However, intracerebral hemorrhage can occur even during treatment with direct oral anticoagulants. The newly developed idarucizumab for direct thrombin inhibitor or andexanet for factor Xa inhibitors can be used for immediate reversal of the anticoagulant effects of direct oral anticoagulants (Pollack et al., 2017; Connolly et al., 2019).

### Platelet Transfusion for Intracerebral Hemorrhage Associated With Antiplatelet Use

The situation regarding reversal of antiplatelet effects is not yet sufficiently advanced. In an open-label phase 3 trial that compared platelet transfusion plus standard care to standard care alone for intracerebral hemorrhage associated with antiplatelets use, 97 participants were randomly assigned to receive platelet transfusion and 93 to receive standard care between 2009 and 2015. Death or dependence at 3 months was more frequent in the platelet transfusion group (72%) than in the group with standard care alone (56%), and the rate of serious adverse events was higher in patients receiving platelet transfusion (42%) than in those without platelet transfusion (24%). Platelet transfusion seems inferior to standard care for intracerebral hemorrhage associated with antiplatelet use. Platelet transfusion cannot be currently recommended for this indication (Baharoglu et al., 2016).

## Early Hemostatic Therapy

Basically, the coagulation process begins with vessel wall injury, leading to tissue factor expression by activated endothelial cells. Circulating factor VII binds to tissue factor and is converted to factor VIIa to form the tissue factor-factor VIIa complex, which advances the coagulation cascade in collaboration with other coagulation factors to produce thrombin, a potent serin protease. Thrombin rapidly converts plasma fibrinogen to fibrin monomer and factor XIII to factor XIIIa, a transglutamase that covalently cross-links fibrin monomers to yield stable fibrin polymer. Thrombin also plays a role in converting plasminogen into plasmin and thus contributes to not only clot formation, but also clot dissolution, because the hemostatic process is integrated with clot dissolution by plasmin to maintain hemostatic balance (Hudnall, 2012). Tranexamic acid, a synthetic derivative of the amino acid lysine, enters the extracellular space and reversibly attaches to plasminogen via its lysine binding site for fibrin, resulting in inhibition of clot fibrinolysis (Nadeau et al., 2015). Tranexamic acid therefore has the potential to reduce hematoma growth by facilitating clot stabilization. The Tranexamic acid for hyperacute primary IntraCerebral Hemorrhage (TICH-2) trial randomized 2,325 patients with acute intracerebral hemorrhage to receive treatment with either tranexamic acid or placebo. The resulting rate of hematoma expansion, defined as an increase of >6 mL or growth of >33%, was lower in the tranexamic acid group (25%) than in the placebo group (29%, *P* = 0.0300). The tranexamic acid group also showed a lower rate of serious adverse events than the placebo group. However, functional status at 90 days after intracerebral hemorrhage did not differ between patients who received tranexamic acid and those who received placebo, probably due to the small reduction in hematoma enlargement (1.37 mL smaller in the tranexamic acid group than in the placebo group; Sprigg et al., 2018). The small reduction in hematoma enlargement between treatment groups in TICH-2 may be increased if patients with known ongoing intracerebral bleeding is recruited, and CT angiography contrast extravasation, the so-called spot sign, can serve as a biomarker for identifying patients with ongoing cerebral hemorrhage (Goldstein et al., 2007; Wada et al., 2007; Demchuck et al., 2012). In the Spot sign and Tranexamic acid On Preventing ICH growth-AUStralasia Trial (STOP-AUST), 100 patients with spot sign were randomly assigned to the tranexamic acid group (*n* = 50) or the placebo group (*n* = 50). The rate for hematoma expansion was not different between the two groups: 44% in the tranexamic acid group and 52% in the placebo group (*P* = 0.41; Meretoja et al., 2020). Tranexamic acid safely reduced hematoma expansion both in TICH-2 and STOP-AUST, but the hemostatic effects would be insufficient to be translated into improved functional outcomes. In this context, recombinant activated factor VII (rFVIIa) is a candidate for addition to the armamentarium against hematoma enlargement because of its effects on enhancing the hemostatic process.

### Recombinant Activated Factor VII

**FIGURE 1:**
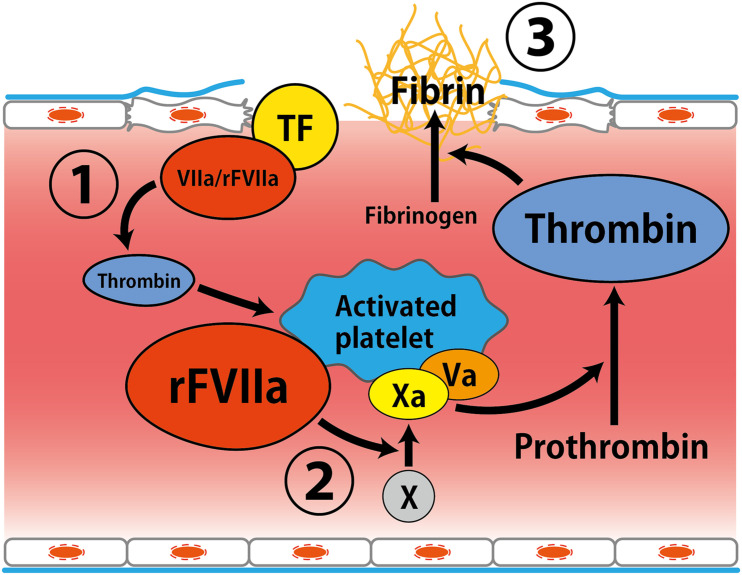
Putative mechanism of hemostatic action by recombinant activated factor VII. (1) Factor VIIa interacts with tissue factor expressed by activated endothelial cells at the site of vessel wall injury, leading to thrombin generation. (2) Amplification of thrombin formation by rFVIIa on the surface of activated platelets. (3) Thrombin converts fibrinogen to fibrin (Monroe et al., 1997; Hoffman and Monroe, 2001). Abbreviations: TF, tissue factor; rFVIIa, recombinant activated factor VII.

Recombinant activated factor VII has been developed as a hemostatic drug for the treatment of hemophilia patients and facilitate hemostasis during spontaneous and surgical bleeds in these patients (Monroe et al., 1997; Roberts, 2004). Although the mechanism of action of rFVIIa has not been clearly established, rFVIIa is suggested to directly activates factor X on the surface of activated platelets, resulting in acceleration of coagulation (Figure 1; Monroe et al., 1997; Hoffman and Monroe, 2001). It has been indicated that rFVIIa promotes hemostasis in a variety of bleeding situations in patients without hemophilia (Roberts, 2004).

The Factor Seven for Acute Hemorrhagic Stroke (FAST) trial, a phase 3 study that compared doses of 80 μg/kg and 20 μg/kg rFVIIa with placebo in 841 patients within 4 h after the stroke onset, showed a significant reduction in hematoma growth by rFVIIa treatment. The mean estimated increase in volume of the hematoma at 24 h was 26% in the placebo group, as compared with 18% in the 20 μg/kg rFVIIa group (*P* = 0.09) and 11% in the 80 μg/kg rFVIIa group (*P* < 0.001). The growth in volume of hematoma was reduced by 2.6 mL in the 20 μg/kg rFVIIa group (*P* = 0.08) and by 3.8 mL in the 80 μg/kg rFVIIa group (*P* = 0.009), as compared with the placebo group. The preventing effect on hematoma growth was particularly large in the group receiving 80 μg/kg of rFVIIa, clearly greater than the preventive effect of tranexamic acid. However, the arterial thromboembolic serious adverse events were more frequent in the group receiving 80 μg/kg rFVIIa than in the placebo group (4% in the placebo group, 5% in the 20 μg/kg rFVIIa group, and 8% in the 80 μg/kg rFVIIa group), and no significant difference in the proportion of patients with severe disability or death was demonstrated (24% in the placebo group, 26% in the 20 μg/kg rFVIIa group, and 29% in the 80 μg/kg rFVIIa group; Mayer et al., 2008). Nevertheless, a *post hoc* analysis of the FAST trial suggests a benefit of rFVIIa in a target subgroup comprising patients ≤70 years with baseline hematoma volume <60 mL, intraventricular hemorrhage volume <5 mL, and time from onset-to-treatment ≤2.5 h. In this target subgroup, the reduction volume in hematoma growth by treatment with the 80 μg/kg rFVIIa was even greater, at 7.3 mL (Mayer et al., 2009). To determine whether younger patients with intracerebral hemorrhage without extensive bleeding at baseline can benefit from 80 μg/kg of rFVIIa given earlier from the stroke onset, the rFVIIa for Acute Hemorrhagic Stroke Administered at Earliest Time (FASTEST) trial is now being prepared to determine this potential benefit of rFVIIa (ClinicalTrials.gov, 2018). The trial includes subjects who are able to be treated with 80 μg/kg rFVIIa within 120 min of stroke onset, with a baseline hematoma volume of <60 mL, no or a small volume of intraventricular hemorrhage, and age ≤80 years (Broderick et al., 2021). Bodies of the United States, Japan, Germany, United Kingdom, Spain, and Canada are planning to participate in this trial, reflecting the pressing need to develop therapeutic strategies against hematoma enlargement, a powerful but modifiable prognostic factor in patients with intracerebral hemorrhage.

## Conclusion

At present, treatments with sufficient outcome-improving effects for patients of intracerebral hemorrhage have not yet been demonstrated, but the recent evolutionary success of mechanical thrombectomy trials for acute ischemic stroke highlights the importance of working to improve appropriate patient selection (Goyal et al., 2016; Albers et al., 2018; Nogueira et al., 2018). In the area of acute ischemic stroke, the effectiveness of neuroprotection and stem cell therapy is currently being actively investigated as a potential adjunct to thrombolysis or mechanical thrombectomy (Hill et al., 2020). The same situation can be expected as a future scene for patients with intracerebral hemorrhage. Treatment strategies for secondary brain damage after intracerebral hemorrhage will become even more important once effective treatments are established for early hematoma expansion.

## Author Contributions

KTa contributed to drafting the article. KTo contributed to critical revision of the article for important intellectual content. Both authors contributed to the article and approved the submitted version.

## Conflict of Interest

The authors declare that the research was conducted in the absence of any commercial or financial relationships that could be construed as a potential conflict of interest.

## Publisher’s Note

All claims expressed in this article are solely those of the authors and do not necessarily represent those of their affiliated organizations, or those of the publisher, the editors and the reviewers. Any product that may be evaluated in this article, or claim that may be made by its manufacturer, is not guaranteed or endorsed by the publisher.
